# Genome-Wide Identification of MKK Gene Family and Response to Hormone and Abiotic Stress in Rice

**DOI:** 10.3390/plants13202922

**Published:** 2024-10-18

**Authors:** Fan Zhang, Jingjing Wang, Yiwei Chen, Junjun Huang, Weihong Liang

**Affiliations:** 1 College of Life Sciences, Henan Normal University, Xinxiang 453007, China; 18236340771@163.com (F.Z.); jjwang202306@163.com (J.W.); yiweichen2025@163.com (Y.C.); 2 The Observation and Research Field Station of Taihang Mountain Forest Ecosystems of Henan Province, Xinxiang 453007, China

**Keywords:** T2T-NIP genome, OsMKK, hormone, abiotic stress

## Abstract

Mitogen-activated protein kinase (MAPK/MPK) cascades are pivotal and highly conserved signaling modules widely distributed in eukaryotes; they play essential roles in plant growth and development, as well as biotic and abiotic stress responses. With the development of sequencing technology, the complete genome assembly of rice without gaps, T2T (Telomere-to-Telomere)—NIP (version AGIS-1.0), has recently been released. In this study, we used bioinformatic approaches to identify and analyze the rice MPK kinases (MKKs) based on the complete genome. A total of seven OsMKKs were identified, and their physical and chemical properties, chromosome localization, gene structure, subcellular localization, phylogeny, family evolution, and cis-acting elements were evaluated. OsMKKs can be divided into four subgroups based on phylogenetic relationships, and the family members located in the same evolutionary branch have relatively similar gene structures and conserved domains. Quantitative real-time PCR (qRT-PCR) revealed that all *OsMKKs* were highly expressed in rice seedling leaves. The expression levels of all *OsMKKs* were more or less altered under exogenous hormone and abiotic stress treatments, with *OsMKK1*, *OsMKK6*, and *OsMKK3* being induced under almost all treatments, while the expression of *OsMKK4* and *OsMKK10-2* was repressed under salt and drought treatments and IAA treatment, respectively. In this study, we also summarized the recent progress in rice MPK cascades, highlighted their diverse functions, and outlined the potential MPK signaling network, facilitating further studies on *OsMKK* genes and rice MPK cascades.

## 1. Introduction

The MPK (Mitogen-Activated Protein Kinase) cascade is a highly conserved signaling pathway involved in regulating a wide range of cellular processes, including proliferation, differentiation, stress responses, and immune responses to environmental stimuli. It is a convergent signaling pathway that transmits signals from the cell surface to the nucleus, where they regulate gene expression [1]. A classic MPK cascade consists of three key conserved kinases, including an MAPK kinase kinase (MAPKKK or MEKK or MKKK), an MAPK kinase (MAPKK or MEK or MKK), and an MAPK (MPK, extracellular signal-regulated kinase [ERK]). They are phosphorylated sequentially, that is, the activated MKKK phosphorylates and activates MKK, the activated MKK phosphorylates MPK, and the activated MPK then directly acts on downstream targets, including transcription factors and enzymes [2,3,4,5].

MKKs act as a critical node in the MPK cascade [2]; the same MKK protein can be activated by different MKKKs and then activates multiple MPK proteins. However, compared with the MKKK and MPK families, the MKK family has the least number of members, e.g., 10 members in *Arabidopsis thaliana* [6], 8 in *Oryza sativa* [7], 6 in *Brassica oleracea* [8], 10 in *Fagopyrum tataricum* [9], 6 in *Cucumis sativus* [10], and 9 in *Malus domestica* [11], approximately half of that of the MPK family members. The main reason is that the same MKK protein can be activated by different MKKKs and can also catalyze multiple MPK proteins. The structure of *MKK* genes is broadly conserved. MKKs can be divided into five subgroups (Groups A–E) based on their structures. Each group contains the activation T loop (S/T-X_3–5_-S/T) (X is any amino acid); the two conserved serine (S) and threonine (T) residues that are phosphorylated by MKKK; an ATP binding signature, consisting of the P loop consensus sequence (GxGxxG) and consensus docking domain (CDD); and the catalytic C loop, consisting of the DΨK consensus [10,12]. More specifically, Group B MKKs have a unique C-terminal NTF2 (Nuclear transport factor 2) domain, which may be involved in nuclear localization.

A growing body of research indicates that MKK in plants is integral not only to various biotic and abiotic stress responses but also to multiple facets of plant growth and development, such as gametogenesis, embryogenesis, morphogenesis, senescence, abscission, fertilization, and seed formation [13,14]. For instance, in *Arabidopsis*, MKK9 plays a role in mediating the balance between mitochondrial dysfunction and growth [15]. Additionally, the MKK6–MPK4 signaling pathway is implicated in the cytoplasmic division during the meiosis of microsporocytes [16]. Furthermore, the MKK4/5–MPK3/6-WRKY2/34 pathway regulates the biosynthesis of liposomes during pollen maturation by regulating the expression of *GPT1* (Glucose-6-Phosphate/Phosphate Translocator 1) [17]. The activation of the GhMKK6–GhMPK4 cascade can enhance the resistance of cotton to *Fusarium oxysporum* f. sp. *Vasinfectum* [18]. The PeMKK2a enhances the salt tolerance of populous [19]. Overall, MPK signaling is essential for orchestrating plant responses to environmental cues, coordinating growth and development, and ensuring plant survival and adaptation in diverse ecological niches.

Rice (*Oryza sativa*) serves as a staple food for over half of the global population, playing a crucial role in ensuring global food security [20]. With advancements in sequencing technology, Chinese researchers have published the complete assembly of the rice (*Oryza sativa* ssp. *japonica* cv. Nipponbare) reference genome, designated as T2T (Telomere-to-Telomere)—NIP (version AGIS-1.0) (http://www.ricesuperpir.com/web/nip, accessed on 10 March 2023) [21]. This milestone represents the first instance of achieving a true whole genome assembly without gaps in rice, thereby providing a remarkable theoretical foundation for molecular breeding research in this essential crop. On the basis of the integrity and accuracy of T2T-NIP, we reanalyzed the MKK gene family and their expression under various stress treatments. We also summarized the research progress in rice MKKs in recent years, providing a strong theoretical and experimental basis for further research.

## 2. Results

### 2.1. Identification, Classification, Protein Characteristics, and Chromosome Locations of OsMKK Genes

A total of seven putative *OsMKK* sequences were identified from the complete assembly of the rice Nipponbare reference genome (T2T-NIP, vAGIS-1.0) [20]. They were respectively named *OsMKK1*~*OsMKK10-2,* according to the naming convention for *AtMKK* genes in *Arabidopsis*. The basic information of OsMKKs, including the gene site, protein length, gene ID in different databases, molecular weight (kDa), amino acid composition, isoelectric point (pIs), and subcellular localization, is listed in Tables S1 and S2. The proteins range from 339 aa (OsMKK10-2) to 523 aa (OsMKK3) and a molecular weight of 36.38 to 58.43 kDa. OsMKK10-1 was predicted to be localized to the mitochondrion, and two OsMKKs (OsMKK3 and OsMKK6) were predicted to be localized to the cytoplasm, while the remaining OsMKKs were predicted to be localized to the nucleus (Table S1). The nonpolar amino acid content of all OsMKK protein sequences is approximately 42%, except for OsMKK10-2, which contains 51.9%. Especially, the content of Trp (W) is low in all proteins (Table S2).

**Figure 1:**
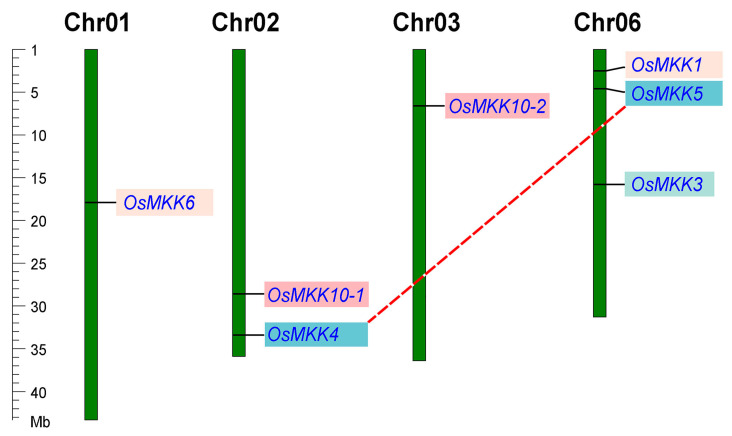
Chromosomal distribution of *OsMKK* genes in rice. The duplicated *OsMKK* genes are shown in red dashed line. Shadows of the same color belong to the same group.

All *OsMKKs* are unevenly distributed on the rice chromosomes. Among them, three *OsMKKs* are located on chromosome 6. Chromosomes 1 and 3 each contain one *OsMKK*, while chromosome 2 contains two *OsMKKs* (Figure 1); no *OsMKK* gene is found on any of the other eight chromosomes. Gene duplication only has two types: segmental duplication and dispersed duplication. Except for *OsMKK4* and *OsMKK5,* which were derived from segmental duplication, the other mapped *OsMKK* genes all resulted from dispersed duplication (Figure 1).

### 2.2. Evolutionary Tree Analysis of MKK Genes from Different Species

**Figure 2:**
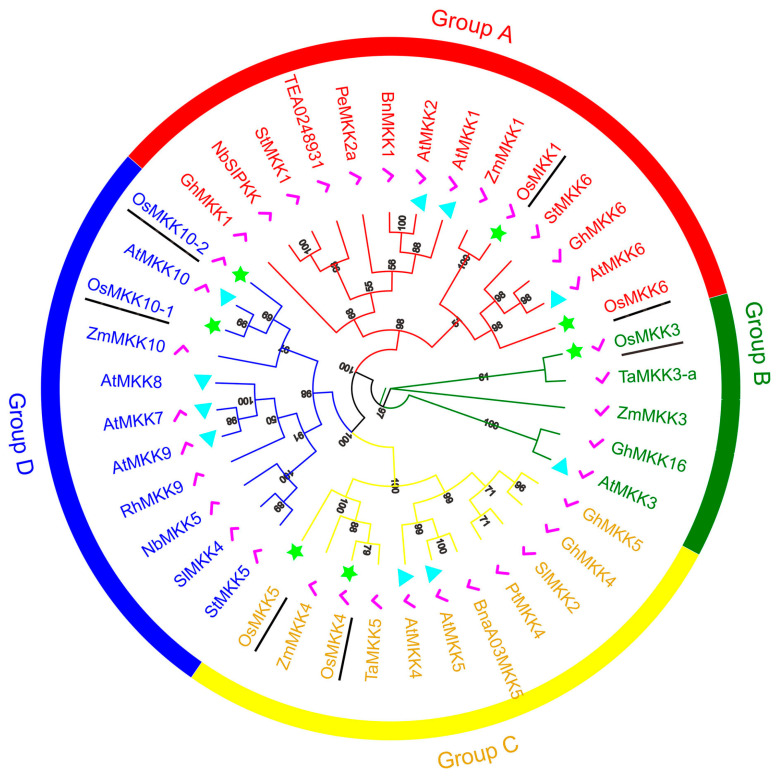
Phylogenetic tree of the 7 OsMKK proteins and 33 known functional MKK proteins from other plants. Green stars represent OsMKKs, cyan triangles represent AtMKKs, and pink checkmarks are known function MKKs.

A phylogenetic tree was constructed using 33 known functional MKK proteins from *Arabidopsis*, rape (*Brassica napus*), cotton (*Gossypium hirsutum*), tobacco (*Nicotiana benthamiana*), poplar (*Populus deltoides* × *P. euramericana* cv. ‘Nanlin895’), black cottonwood (*Populus trichocarpa*), tomato (*Solanum lycopersicum*), rose (*Rosa hybrida*), potato (*Solanum tuberosum*), wheat (*Triticum aestivum*), tea plant (*Camellia sinensis*), and maize (*Zea mays*), and all OsMKK proteins from rice (Table S3) to enhance the understanding of molecular evolution and phylogenetic relationship of plant MKK proteins. The OsMKK proteins could be classified into four subgroups (Groups A–D) based on the analysis of sequence homology and phylogeny, and Group B showed unique functions related to development and drought stress (Figure 2 and Table S3). It is suggested that the functions of rice MKK proteins may primarily involve abiotic stress, immunity, and development, similar to those observed in other species.

### 2.3. Gene Structure, Conserved Motifs, and Three-Dimensional Domain Analysis

**Figure 3:**
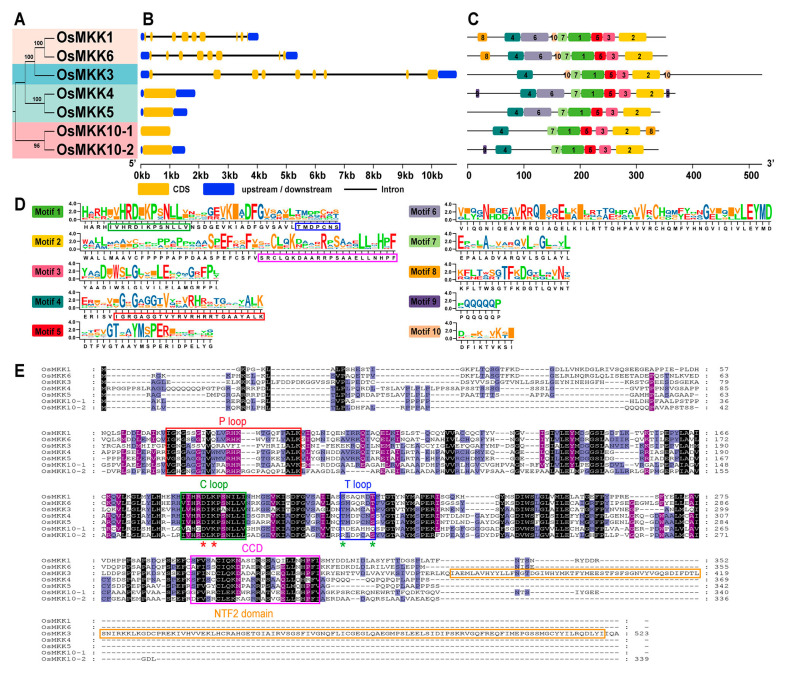
Gene structure and conserved protein motifs analysis of *OsMKK* genes. (**A**) ML phylogenetic tree analysis of OsMKKs. (**B**) Exon–intron structure of *OsMKKs*, where golden yellow boxes represent coding sequences (CDS), the blue boxes represent upstream/downstream sequences, and the black lines represent the introns. (**C**) The conserved motifs in OsMKK proteins. The ten conversed motifs are displayed in various unique colors. The gene and protein length are indicated by the scale at bottom. (**D**) Sequence logos of ten conserved domains. The conserved sequences of the different motifs are highlighted in different colored rectangles. (**E**) Sequence alignment and motif analysis of OsMKKs. Identical amino acids are shaded black, and similar amino acids are shaded purple. The P, C, and T loops, CCD, and the NTF2 domain are highlighted in colored rectangles (P loop: red; C loop: green; T loop: blue; CDD: pink; NTF2: yellow). The red stars show the active site, and the green stars indicate the phosphorylation site of OsMKK proteins. Species information can be found in Figure S1.

The gene structures were ordered in accordance with the phylogenetic tree, with considerable similarity observed within the same group (Figure 3A). Groups A and B have approximately eight exons each, while groups C and D only have one exon (Figure 3B). Except for *OsMKK10-1*, all other genes contain 5′ UTR and 3′ UTR regions (Figure 3B).

**Figure 4:**
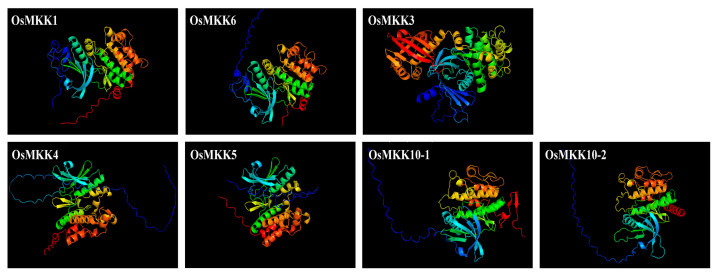
Predicted 3D models of OsMKK proteins. Models have been generated by Alpha 2 and visualized by rainbow color from N (blue) to C terminus (red) using PyMOL v2.5.8 software.

Conserved motif analysis of OsMKK proteins reveals that motifs within the same group share great similarities (Figure 3C,D and Figure S1). The three-dimensional structures of OsMKK proteins predicted by Alpha2 also show that MKK in the same group are similar in protein structure (Figure 4). Moreover, each OsMKK has six conserved motifs, arranged as motif 4, motif 7, motif 1, motif 5, motif 3, and motif 2 (Figure 3C), suggesting functional similarity among the OsMKK family members. Additionally, different groups exhibit motif diversity, e.g., motif 10 is only found in groups A and B, motif 6 in groups A and C, motif 9 in groups C and D, and motif 8 in groups A and D (Figure 3C), indicating potential functional differentiation. The OsMKKs alignment and conserved domain analysis showed that all the OsMKKs contained the T loop, the C loop, the P loop, and the CDD, located on motif 1, motif 1, motif 4, and motif 2, respectively (Figure 3D,E). Moreover, OsMKK3 in group B had a unique C-terminal NTF2-like domain, which may be essential for its nuclear localization (Figure 3E).

### 2.4. Expression Pattern of OsMKK Genes in Different Tissues

**Figure 5:**
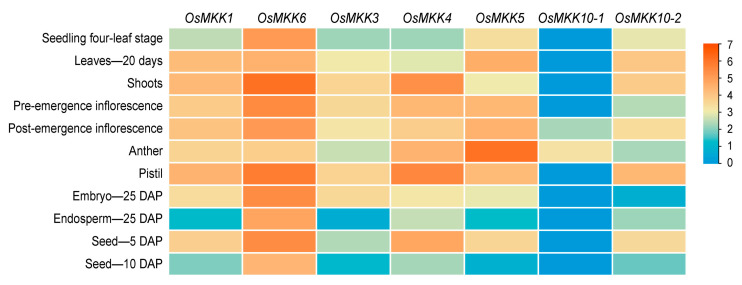
Expression profiling of 7 *OsMKK* genes in different organs and tissues. The red color represents high-level expression, while the blue color represents low-level expression. DAP, days after pollination.

Expression pattern analysis helps predict the biological function of genes. The spatiotemporal functions of *OsMKKs* were studied on the basis of transcriptome data from 11 different tissues, including shoots, anther, pistil, leaves—20 days, embryo—25 DAP (Days After Pollination), endosperm—25 DAP, seed—5 DAP, seed—10 DAP, pre-emergence inflorescence, post-emergence inflorescence, and seedling four-leaf stage. As shown in Figure 5, the *OsMKK* genes within the same group showed similar expression patterns, particularly *OsMKK4/OsMKK5*. Notably, during the vegetative growth phase of rice, all *OsMKK* genes except for *OsMKK10-1* were highly expressed in the leaves, while a considerable difference in gene expression was observed during the reproductive stage, with *OsMKK6* being highly expressed and *OsMKK10-1* showing low-level expression. The results indicated that the *OsMKK* genes in rice may play specific biological functions in the growth and development of various tissues.

**Figure 6:**
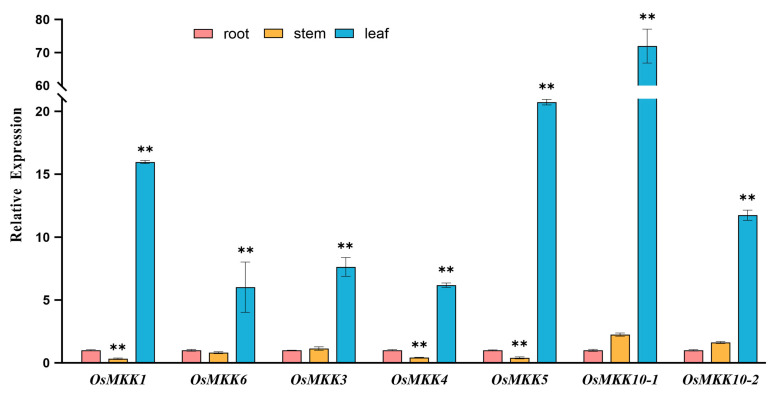
Expression patterns of 7 *OsMKK* genes in the roots, stems, and leaves of rice seedlings. Data are represented as the mean ± SD of three independent replicates. Asterisks indicate statistically significant differences compared with root (** *p* < 0.01; Student’s *t*-test).

We also analyzed the expression patterns of *OsMKKs* in the roots, stems, and leaves of rice seedlings by qRT-PCR. Similarly, the expression profiles of the *OsMKK* genes within the same group were similar (Figure 6). *OsMKK1*, *OsMKK4*, and *OsMKK5* showed low expression in the stems and high expression in the leaves. The remaining genes showed almost no difference in expression between the root and stem and were still highly expressed in leaves. While group A and C gene numbers had the lowest expression levels in the stems of rice seedlings, group B and D gene numbers had the lowest expression levels in the roots; all *OsMKK* gene members were generally highly expressed in the leaves (Figure 6).

### 2.5. Identification of Cis-Acting Elements in OsMKKs’ Predicted Promoters

**Figure 7:**
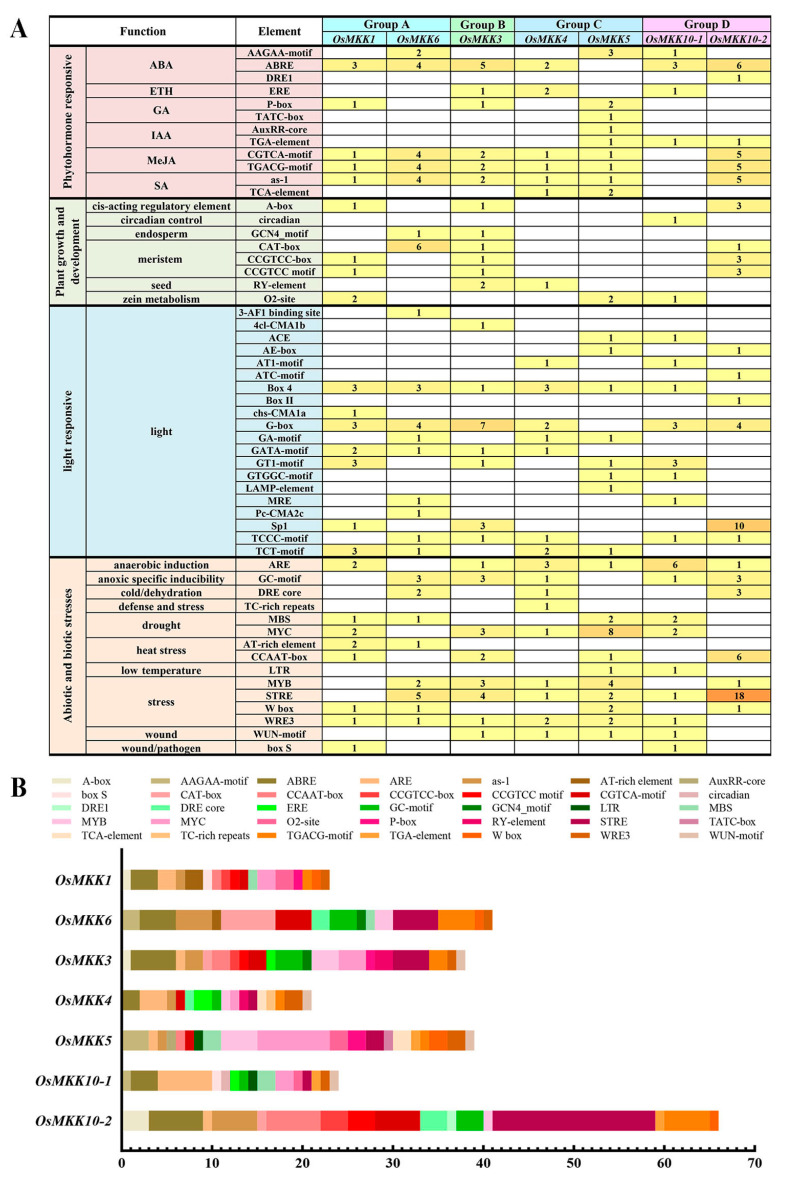
Cis-acting elements in the promoter of *OsMKK* genes. (**A**) Numbers of predicted cis-acting elements in *OsMKK* promoters are shown. (**B**) The distribution of predicted cis-acting elements on different gene promoters. Different colors represent different cis-acting elements.

The upstream 2 kb sequences of the initiation codons of the *OsMKKs* were uploaded to the PlantCARE database to identify putative cis-acting elements and further investigate the potential regulatory functions of *OsMKKs*. A total of 346 cis-acting elements were found among all *OsMKKs* and clustered into four subdivisions: hormone-response elements, plant growth and biological processed elements, light-responsive elements, and stress-response elements (Figure 7 and Table S4). Among the four types, stress-associated elements accounted for the largest proportion (~37.6%), followed by light-responsive elements and hormone-response elements; plant growth and biological processed elements (~9.8%) were the least. Each *OsMKK*’s promoter contained more than 11 stress-associated elements. Approximately 34% of the 88 plant hormone-response elements were involved in abscisic acid (ABA) responsiveness, existing in all *OsMKKs*. Except for *OsMKK10-1*, every *OsMKK* had methyl jasmonate (MeJA)-responsive and salicylic acid-responsive elements (Figure 7A,B).

### 2.6. Expression Pattern of OsMKKs Under Hormone and Abiotic Stresses

**Figure 8:**
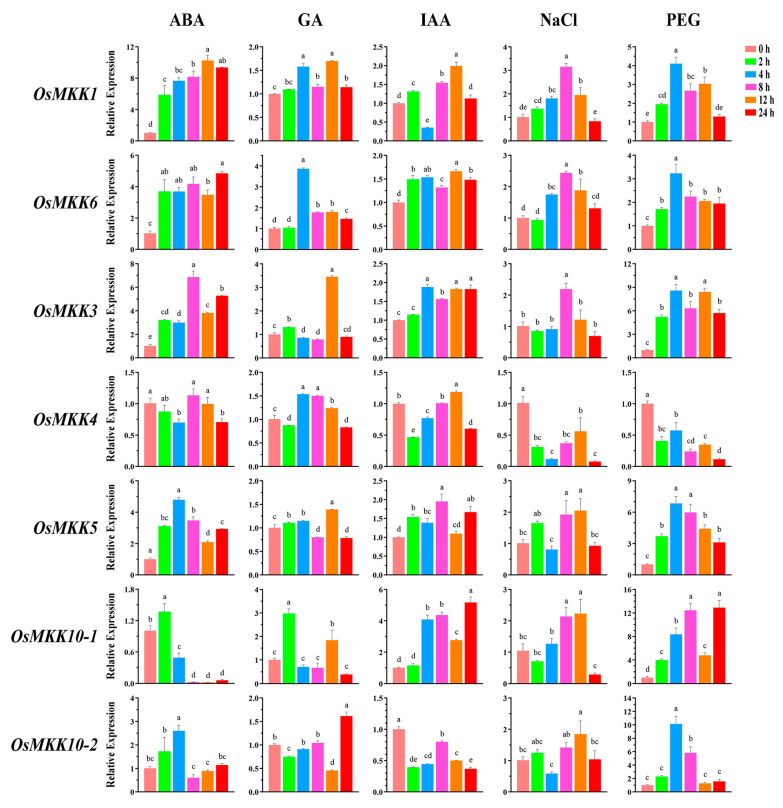
Expression levels of *OsMKK* genes under ABA, GA, IAA, salt, and drought stress treatments. Data are represented as the mean ± SD of three independent replicates. Different letters above bars indicate significant differences (*p* < 0.05; Tukey’s test).

Promoter analysis revealed that *OsMKK* genes may be related to plant stress and hormone responses, and the expression patterns of all *OsMKKs* under five treatments were verified by qRT-PCR (Figure 8). After ABA treatment, the expression levels of *OsMKK1*, *OsMKK6*, *OsMKK3*, and *OsMKK5* were upregulated; *OsMKK4* was first downregulated, then upregulated, and then downregulated again; and *OsMKK10-1* and *OsMKK10-2* were first upregulated and then downregulated. After GA treatment, the expression levels of all *OsMKKs* were more or less upregulated except for the expression levels of *OsMKK10-1* and *OsMKK10-2*, which were fluctuating greatly. After IAA treatment, *OsMKK1* was first upregulated, then downregulated, and then upregulated again. *OsMKK4* and *OsMKK10-2* were downregulated, while the remaining genes were upregulated. Under salt stress and drought treatments, the expression levels of almost all genes were upregulated, except for *OsMKK4*. These results indicate that *OsMKK4* may play negative roles under salt and drought treatments, whereas other genes may play positive roles under hormone and abiotic stress treatments.

## 3. Discussion

The MPK pathway is found in all eukaryotes and is a highly conserved plant cell signaling pathway that plays an important role in plant growth, development, and stress response [19,22,23,24]. Compared with MPK and MKKK, the MKK gene family contains the fewest number of genes and has been identified in previous studies in different organisms, such as *Carya illinoinensis* (4 genes), *Prunus mume* (7 genes), *Setaria italica* (10 genes), *Homo sapiens* (7 genes), and so on [25,26,27,28]. They often contain the same conserved domain; however, the activation T loop is S/T-X_3_-S/T in animals and S/T-X_5_-S/T in plants. Tandem and segmental duplications have long been proposed as the main contributors to the gene family expansion in plants [29]. However, our results showed that dispersed duplication events were the primary reason and that no tandem duplication events occur in the *OsMKK* gene family (Figure 1), suggesting why few *OsMKK* genes have been identified.

Nearly 20 years ago, a total of eight OsMKKs were identified in the rice genome [7]. However, systematic research on them has yet to be reported. With the release of the latest telomere-to-telomere gap-free reference genome of rice [21], we conducted a thorough analysis of MKK family genes and identified seven *OsMKKs* from the rice T2T-NIP genome. Compared with the previous studies, the *OsMKK* genes are the same as those reported in rice, except for *OsMKK10-3* [7]. The *OsMKK10-3* does not exist in the latest annotated rice T2T-NIP genome (Table S1). Previous studies have pointed out that the issue of *OsMKK10-3*′s functionality might be largely moot and confirmed that no protein interacted with it using experimental approaches (in vivo and in vitro) [7,30]. Perhaps, given the incomplete and inaccurate annotation of rice genes in the past, people have always believed that eight *OsMKK* genes exist, but in reality, only seven exist. We also tested the expression levels of *OsMKK10-3* and observed no expression. Therefore, it is highly likely that *OsMKK10-3* does not exist. The T2T-NIP genome of rice only has seven *OsMKK* genes.

**Figure 9:**
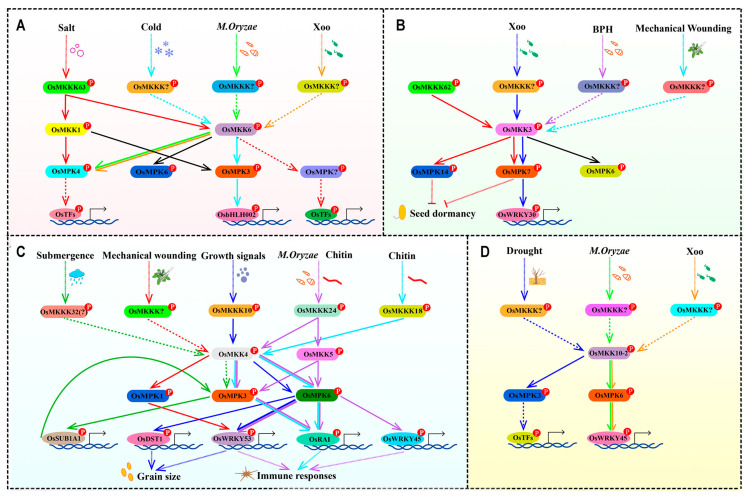
OsMKKs are involved in plant growth and development and diverse biotic and abiotic stresses. (**A**–**D**) represent the processes in which each subgroup participates [3,35,36,39,40,41,42,45,46,47,48,49,50,51,52,53,54,55].

Through multiple sequence alignment and phylogenetic analysis, OsMKKs are classified into four groups based on the evolution of the S/T-X_5_-S/T conserved motifs and functions (Groups A–D) (Figure 2). OsMKK1 and OsMKK6 belong to group A along with MKK from other plants with known functions, such as ZmMKK1 [31], AtMKK6 [32,33], GhMKK6 [18], and StMKK6 [34]. ZmMKK1 is associated with drought and salt tolerance in transgenic *Arabidopsis* [31]. The OsMKKK63-OsMKK1-OsMPK4 cascade confers tolerance to salt stress [35,36] (Figure 9A). OsMKK1 plays roles in triggering downstream stress-responsive pathways and may be involved in drought stress because OsMKK1 and ZmMKK1 are on the same branch (Figure 2). AtMKK6 regulates cytokinesis through the downstream target protein AtMPK4 [32,33]. The GhMKK6–GhMPK4 cascade functions downstream of the scaffold protein GhMORG1 and confers resistance to cotton against *Fusarium oxysporum* [18]. StMKK6 plays an important role in potato immunity, while the OsMKKK63–OsMKK6 and OsMKK6–OsMPK3 cascades are involved in seed dormancy and cold tolerance, respectively [34,35,37,38]. Thus, OsMKK6 may play roles in innate immunity and disease resistance. As shown in Figure 9B, OsMKK3 is related to Xoo, BPH, seed dormancy, and mechanical wounding and may also be related to drought and salt stress according to evolutionary clues (Figure 2) [39,40,41,42,43,44]. The OsMKK4 and OsMKK5 in Group C have been extensively studied, and their functions are relatively clear, while in Group D, only OsMKK10-2 has been studied (Figure 9C,D) [3,45,46,47,48,49,50,51,52,53,54]. OsMKK10-1 may have functions in drought tolerance and defense responses, or OsMKK10-1 may have a weakened function because of gene inactivation due to its low expression (Figure 5). Therefore, further research is needed to investigate its function.

As mentioned above, *OsMKK* genes in rice play diverse roles in signaling pathways that regulate stress responses, growth, development, and defense mechanisms. Studying these genes can provide valuable insights into the molecular mechanisms underlying plant responses to environmental challenges and help in the development of stress-tolerant and high-yielding rice varieties. However, so far, only a few complete MPK cascades have been confirmed, such as the OsMKKK63–OsMKK1–OsMPK4 cascade, OsMKKK62–OsMKK3–OsMPK7/14 cascade, OsMKKK10–OsMKK4–OsMPK6 cascade, OsMKKK18–OsMKK4–OsMPK3/6 cascade, and OsMKKK24–OsMKK4/5–OsMPK3/6 cascade (Figure 9) [3,35,36,39,45,46,49,55]. Therefore, the minimum number of OsMKKs that play bridging roles in the MPK cascade in rice must be studied in subsequent research.

Gene expression and function are closely related to gene structure and can also provide information for studying the evolution of gene families [54,56]. Our results showed that *OsMKK* genes from groups A and B contained seven to eight introns, while the other two subgroups (groups C and D) had no introns (Figure 3B), indicating that the loss or gain of introns may be an important reason for the gene functional differentiation of *OsMKKs*. We also identified ten highly conserved motifs in OsMKKs, including their three-dimensional protein structure (Figure 3C–E, Figure 4, and Figure S1), and found that the OsMKKs from the same group had similar structures but were not completely identical, indicating that they might have similar or different functions. The changes in conserved motifs allow proteins to be classified into subfamilies and reflect the specific functions of each subfamily [57]. Interestingly, the expression levels of all *OsMKKs* in leaves were higher than those in other tissues (Figure 6). This phenomenon might occur because roots consist of ~90% mature cells, and stems mainly have a transport function and consist of 90% cells with condensed chromatin. The only tissue with a high metabolic activity and a high level of gene expression is the leaf. Therefore, single-cell sequencing must be conducted in subsequent experiments to understand the expression of *OsMKKs* further.

According to previous studies, the functions of *OsMKK* are mainly divided into the following categories: plant stress resistance, plant growth and development, and hormone signaling [13,14]. The identification of cis-acting elements in promoters is vital for understanding the molecular switches controlling gene activity in different biological processes, including developmental processes, hormone responses, and abiotic stress responses [58]. Our comprehensive analysis for identifying and characterizing cis-acting elements within the *OsMKK* promoter sequence provides an improved understanding of the regulation of *OsMKK* genes. All OsMKKs contain relatively more hormone and stress response elements (Figure 7 and Table S4). A relationship between hormones and stress responses exists. For example, previous studies have proven that MeJA is not only involved in plant signaling but also alleviates different stresses and counteracts the toxicity of heavy metal stress, low temperature stress, drought stress, salt stress, and pathogenic bacteria [59]. Except for *OsMKK10-1*, all other *OsMKK* genes contain the MeJA response-associated TGACG-motif and CGTCA-motif and the stress-response-associated ARE, STRE, or/and WRE3 (Figure 7). Therefore, we hypothesized that *OsMKKs* respond to abiotic stress through the jasmonate pathway in the leaves and roots. In situ hybridization will be used to investigate stress related to growth/hormone redistribution in the future. In addition, the OsMKK promoter regions harbor a series of other elements responsive to nearly all types of hormones, including ABA, auxin (IAA), salicylic acid (SA), gibberellin (GA), and ethylene (ETH). Previous studies indicated that the MPK cascade has a possible role in the regulation of nitrogen metabolism [60]. However, we did not identify the nitrate-responsive DNA elements in any of OsMKKs. Interestingly, many hormones are related to the absorption and utilization of nitrogen. ABA contributes to the optimization of nitrate uptake by regulating the expression of *NRT2/NAR* in wheat [61]. The DNR1–Auxin–OsARFs cascade enhances crop NUE (nitrogen use efficiency), and the key proteins GRF4 and NGR5 in rice that make them more efficient in utilizing nitrogen fertilizer are key components of the gibberellin signaling pathway [62,63,64]. OsMKKs may be involved in nitrogen utilization through hormone-response elements. In addition, all the *OsMKK* genes were more or less induced/repressed by five different treatments in the experiments (Figure 8). The dissimilar variation trends in the expression levels of *OsMKK* genes at different stages of the same treatment implied that the different *OsMKK* genes had diverse responses to the same treatment. Moreover, auxin and other hormones act locally, and a balance may have occurred between up and down regulation in different cell types (in epidermis and mesophyll, as a hypothetical case), but the total expression remains unchanged. It is also possible that the cascade regulation of expression in different cell types/positions is responsible for different time under various treatments. These results suggest that *OsMKK* genes are crucial for various aspects of growth, development, and responses.

## 4. Materials and Methods

### 4.1. Identification of the OsMKK Gene Family

To identify the *OsMKK* gene family in the rice T2T-NIP genome, we downloaded the nucleotide and protein sequences of rice T2T-NIP from an online database (http://www.ricesuperpir.com/web/nip, accessed on 10 March 2023) and used HMMER 3.0 to identify rice sequences that contained the serine/threonine-protein kinase-like domain (PF00069, E-value < 1 × 10^−5^). Then, the candidate putative *OsMKK* genes were validated by SMART3 and NCBI-CDD (https://www.ncbi.nlm.nih.gov/Structure/bwrpsb/bwrpsb.cgi, accessed on 10 March 2023). The putative OsMKK protein sequences were further confirmed manually for further analysis. The number of amino acids, molecular weight, and isoelectric point of the *OsMKK* gene family were calculated using the ExPASy online website (https://web.expasy.org/protparam/, accessed on 12 March 2023), and subcellular localization was predicted using the online website (https://services.healthtech.dtu.dk/services/DeepLoc-2.0/, accessed on 12 March 2023).

### 4.2. Chromosomal Distribution, Gene Structure, Conserved Motifs, and Three-Dimensional Domain Analysis

Based on information from the T2T-NIP genome, the chromosomal distribution and gene structure of *OsMKK* genes were visualized using MapChart (v2.3.2) and TBtools-II (v2.069) softwares, respectively [65,66]. The conserved motifs of OsMKKs were predicted by MEME Suite v5.5.5 (https://meme-suite.org/meme/tools/meme, accessed on 15 March 2023) and performed by TBtools-II [66]. Highly accurate three-dimensional protein structures of OsMKKs were predicted using the online tool ColabFold v1.5.5 (https://colab.research.google.com/github/sokrypton/ColabFold/blob/main/AlphaFold2.ipynb, accessed on 18 March 2023) and visualized by PyMOL (v2.5.8) software [67].

### 4.3. Phylogenetic Analyses

The protein sequence comparison of OsMKKs and 33 previously reported MKKs from other plant species was performed using MAFFT v7.0 (https://mafft.cbrc.jp/alignment/server/, accessed on 25 March 2023) with default parameters. The results were then used to construct the unrooted phylogenetic tree by MEGA 11 using the Maximum Likelihood (ML) algorithm with 1000 bootstrap replicate. The tree was visualized and edited using Evolview v3 [68]. The duplication of various types (whole-genome duplication, tandem duplication, proximal duplication, segmental duplication, transposed duplication, and dispersed duplication) of OsMKK gene pairs was detected by TBtools-II [66].

### 4.4. Transcriptome Data Source and in Silico Expression Analysis

In order to analyze the expression of *OsMKKs* during rice growth and development, we downloaded the published transcriptome data from the website (http://rice.uga.edu/expression.shtml, accessed on 15 April 2023) and used TBtools-II to create heat maps [66]. The rice tissue-specific expression data consisted of 11 stages, as follows: shoots, anther, pistil, leaves—20 days, embryo—25 DAP (Days After Pollination), endosperm—25 DAP, seed—5 DAP, seed—10 DAP, pre-emergence inflorescence, post-emergence inflorescence, and seedling four-leaf stage (Table S5).

### 4.5. Analysis of Cis-Acting Elements in OsMKKs’ Promoters

The 2000 bp upstream of the translation start site of each *OsMKK* gene was obtained from rice T2T-NIP genome and then submitted to the PlantCARE website (http://bioinformatics.psb.ugent.be/webtools/plantcare/html/, accessed on 3 May 2023) to identify the potential cis-acting elements (Table S4).

### 4.6. Plant Materials and Treatments

The rice (*Oryza sativa* L. var. *japonica* cv. Nipponbare) seedlings were grown in the Yoshida culture solution in the 28 °C growth chamber at Henan Normal University; the light/dark photoperiod was 16/8 h and relative humidity was 60% [69]. The roots, stems, and leaves from the three-leaf/one-heart-period rice plants were respectively collected for RNA extraction. To examine the response of *OsMKKs* to hormone and abiotic stresses, seedlings from three-leaf/one-heart-period were exposed to diverse treatments, such as 150 mM NaCl and 20% PEG6000, and 100 μM abscisic acid (ABA), 100 μM gibberellin acids (GA), or 100 μM auxin (IAA) were sprayed on leaves. The leaves of control and treated plants were harvested at the following five time points: 2, 4, 8, 12, and 24 h after treatment initiation. All the obtained samples were immediately frozen in liquid nitrogen and stored at −80 °C. Each biological replicate, including each treatment and time point, was repeated three times.

### 4.7. RNA Extraction and qRT-PCR Analysis of OsMKKs

Total RNA from each leaf sample mentioned above was extracted using RNAiso Plus* (TaKaRa, Dalian, China) and then reverse transcribed into cDNA with PrimeScript™ FAST RT reagent Kit with gDNA Eraser (TaKaRa). The qRT-PCR was performed using Ace Qpcr SYBR^®^ Green Master Mix (Vazyme, Nanjing, China) following the manufacturer’s instructions. OsAct1 was used as an internal control. The primers for qRT-PCR are listed in Table S6, and 2^−ΔΔCt^ method was used to calculate the relative expression levels of *OsMKKs*.

### 4.8. Statistical Analysis

In this study, all experiments were performed with three independent repetitions. For all assays, quantitative data are reported as means ± standard deviation (SD). One-way ANOVA Tukey’s test (*p* < 0.05) and Student’s *t*-test (* *p* < 0.05; ** *p* < 0.01) were used for statistical analysis. Origin version 8.0 (OriginLab, Northampton, MA, USA) was used to generate the histograms for gene expression levels.

## Data Availability

The data that support the findings of this study are available within the paper and its Supplementary Materials online.

## Supplementary Materials

The following supporting information can be downloaded at https://www.mdpi.com/article/10.3390/plants13202922/s1, Figure S1: Alignment of domains in MKKs of plants. All the MKK protein sequences were aligned by MAFFT v7.0. Sequences of additional MKK domains that are highlighted are ATP binding signature, consisting of the P loop consensus sequence (GxGxxG), marked in cyan; the catalytic C loop, consisting of the DΨK consensus, marked in orange color; the activation T loop, marked in green color; and NTF2 domain, marked in pink color. The sequence derivations from the S/TxxxxxS/T activation loop of Clade C and D are marked in a lighter shade of green color. Table S1: Characteristic features of the *MKK* gene family identified in rice; Table S2: Composition of OsMKK protein; Table S3: The 33 known MKK in plants; Table S4: List of identified CRE and their putative function in 7 *OsMKKs’* promoters; Table S5: Rice tissue-specific expression data; Table S6: Primers used for q-RT expression analysis of 7 *OsMKK* genes.
