# Supplementary material for: Genome-Wide Identification of MKK Gene Family and Response to Hormone and Abiotic Stress in Rice
Source: Plants (Basel). 2024 Oct 18;13(20):2922. doi: 10.3390/plants13202922 (PMC11510841; doi:10.3390/plants13202922)
Supplement: Supplementary file 1 [file plants-13-02922-s001.zip › Fig S1 additional MKK domain.pdf]

Group A

```
                                                                                                                              GxGxxG
OsMKK6       -MRGK-KPHKELKLSVPA-QETPVDKFL---------TASGTFKDGELRLNQRGLQLISEETADEPQSTNLKVE--D-VQLSMDDLEMIQVIGKGSGGIVQLVRHKWVGTLYALKGIQMN 105
AtMKK6       MVKIK-SNLKQLKLSVPA-QESPISSFL---------TASGTFHDGDFLLNQKGLRLTSDEKQSRQSDSKEL----D-FEITAEDLETVKVIGKGSGGVVQLVRHKWVGKFFAMKVIQMN 104
GhMKK6       -MKSK-KPLKQLKLAVPA-QETPISSFL---------TASGTFHDGDLLLNQKGLRLISEEKESRPSDAKEL----D-FEFSLEDLETIKVIGKGSGGVVQLVRHKWVGRLFALKVIQMN 103
StMKK6       -MKTT-KPLKQLKLSVPA-QDTPISSFL---------TASGTFHDGDLLLNQKGLRLISEENESLPSETKEI----D-LQFSLEDLETIKVIGKGSGGVVQLVRHKWVGTLFALKVIQMN 103
OsMKK1       MGKPG-------KLALPS-HESTIGKFL---------TQSGTFKDGDLLVNKDGLRIVSQSEEGEAPPIEPL----DHNQLSLDDLDAIKVIGKGSSGIVQLVRHKWTGQFFALKVIQLN  99
ZmMKK1       ----MATPRKPIKLTLPS-HETTIGKFL---------THSGTFTDGDLRVNKDGLRIVSRREGGEAPPIEPL----D-SQLSLDDLDVIKVIGKGSSGNVQLVRHKFTGQFFALKVIQLN 101
AtMKK1       MNRGSLCPN---PICLPP-LEQSISKFL---------TQSGTFKDGDLRVNKDGIQTVSLSEPGAPPPIEPL----D-NQLSLADLEVIKVIGKGSSGNVQLVKHKLTQQFFALKVIQLN 102
AtMKK2       MKKGGFSNN--LKLAIPVAGEQSITKFLRKGFGSLCRTQSGTFKDGDLRVNKDGVRIISQLEPEVLSPIKPA----D-DQLSLSDLDMVKVIGKGSSGVVQLVQHKWTGQFFALKVIQLN 113
BnMKK1       MKKGGFSNN--LKLSIPPAGEQSITKFL---------TQSGTFKDGDLRVNKDGVRIVSQSEPEALSPIKPA----D-DKLGLSDLDMVKFVGKGSSGVVQLVQHKWTGQFFALKVIQLN 104
PeMKK2a      MKKGGLNPNVKLKLSLPPPKEVSFAKFL---------TQSGTFRDGDLLVNRDGVRIVSQIDTQPPPPITPT----D-NQLSLADIDMVKVIGKGSSGIVQLVQHKWTSQFFALKVIQMN 106
TEA024893.1  MKKGTLRPS--LKLSLPPSDEVAFSKFL---------TESGTFMDGDLLVNRDGVRIVSQSQVETPPLIQPS----D-NQISLADFDAIKVIGKGNGGIVRLVQHKWTGQFFALKVIQMN 104
StMKK1       MKKGSFAPN--LKLSLPPPDEVALSKFL---------TESGTFKDGDLLVNRDGVRIVSQSEVAAPSVIQPS----D-NQLCLADFEAVKVIGKGNGGIVRLVQHKWTGQFFALKVIQMN 104
NbSIPKK      MKKGSFAPN--LKLSLPPPDEVNLSKFL---------TESGTFKDGDLLVNRDGVRIVSQSEVEAPSVIQPS----D-NQLCLADFEAVKVIGKGNGGIVRLVQHKWTGQFFALK---MN 101
GhMKK1       MKKGKGIVNPNLKLSLPPPAELSFAKFL---------TESGTFKDGDLLVNKDGVRIVSESESEAPPPIKPSETDAD-DQLNLDDIDAIKVIGKGNGGIVQLVQHKWTGQFFALKIIQMN 110

                                                                                                                  DΨK
OsMKK6       IQEAVRKQIVQELKINQ-ATQNAHIVLCHQSFYHNGVIYLVLEYMDRGSLADIIKQVKTILEPYLAVLCKQVLEGLLYLHHERHVIHRDIKPSNLLVNRKGEVKITDFGVSAVLASSMGQ 224
AtMKK6       IQEEIRKQIVQELKINQASSQCPHVVVCYHSFYHNGAFSLVLEYMDRGSLADVIRQVKTILEPYLAVVCKQVLLGLVYLHNERHVIHRDIKPSNLLVNHKGEVKISDFGVSASLASSMGQ 224
GhMKK6       IQEEIRKQIVQELKINQ-ASQCSHVVVCYHSFYHNGAISLVLEYMDRGSLADVIRQVNTILEPYLAVVCKQVLQGLVYLHHERHVIHRDIKPSNLLVNHKGEVKITDFGVSAMLASSMGQ 222
StMKK6       IQEDIRKQIVQELKINQ-ASQCPHVVVCYHSFYHNGAISLVLEYMDRGSLVDVIGQLKTILEPYLAVVCKQVLQGLVYLHHERHVIHRDIKPSNLLVNHKGEVKITDFGVSAMLASSMGQ 222
OsMKK1       IQENIRRQIAQELKISL-STQCQYVVACCQCFYVNGVISIVLEYMDSGSLSDFLKTVKTIPEPYLAAICKQVLKGLMYLHHEKHIIHRDLKPSNILINHMGEVKISDFGVSAIIASSSAQ 218
ZmMKK1       IDESIRKQIAKELKINL-STQCQYVVVFYQCFYFNGAISIVLEYMDGGSLADFLKTVKTIPEAYLAAICTQMLKGLIYLHNEKRVIHRDLKPSNILINHRGEVKISDFGVSAIISSSSSQ 220
AtMKK1       TEESTCRAISQELRINL-SSQCPYLVSCYQSFYHNGLVSIILEFMDGGSLADLLKKVGKVPENMLSAICKRVLRGLCYIHHERRIIHRDLKPSNLLINHRGEVKITDFGVSKILTSTSSL 221
AtMKK2       IDEAIRKAIAQELKINQ-SSQCPNLVTSYQSFYDNGAISLILEYMDGGSLADFLKSVKAIPDSYLSAIFRQVLQGLIYLHHDRHIIHRDLKPSNLLINHRGEVKITDFGVSTVMTNTAGL 232
BnMKK1       VDEAIRKPIAQELKINQ-SSQCPYLVTSYQSFYDNGAISLILEYMDGGSLEDFLKSVKTIPESYLSAIFKQVLQGLIYLHHDKHIIHRDLKPSNLLVNHRGEVKITDFGVSTVMTNTAGL 223
PeMKK2a      IEESARKAITQELKINQ-SSQCPYVVMCYQSLYDNGAISIILEYMDGGSLADFLKSVRKISEPYLAAICKQVLKGLLYLHHEKHIIHRDLKPSNLLINHRGEVKITDFGVSAIMQSTSGQ 225
TEA024893.1  IEESARKQIAQELKINQ-SSQCPYVVVCYQSFYDNGAISIILEYMDGGSLADFLKKVKNIPESYLSAICYQVLKGLSYLHHEMHIIHRDLKPSNLLVNHRGEVKITDFGVSAILQGTAGQ 223
StMKK1       IDESMRKHIAQELRINQ-SSQCPYVVICYQSFFDNGAISLILEYMDGGSLADFLKKVKTIPERYLAVICKQVLKGLWYLHHEKHIIHRDLKPSNLLINHRGDVKITDFGVSAVLASTSGL 223
NbSIPKK      IEESMRKHIAQELRINQ-SSQCPYVVISYQSFFDNGAISIILEYMDGGSLADFLKKVKTIPERYLAAICKQVLKGLWYLHHEKHIIHRDLKPSNLLINHIGDVKITDFGVSAVLASTSGL 220
GhMKK1       IEESARKQIAKELKINQ-SSQCPYVVVCYQSFYKNGAISIILEYMDGGSLADFLRKVKSIPEPYLAAICNQVLKGLMYLHHEKHIIHRDLKPSNLLINHRGEVKITDFGVSAIMTSTSGQ 229

OsMKK6       RDTFVGTYNYMAPERISGSSYDYKSDIWSLGLVILECAIGRFPYIPSE-GEGWLSFYELLEAIVDQPPPSAPADQFSPEFCAFISSCIQKDPAERMSASELLNHPFIKKFE-DKDLDLRI 342
AtMKK6       RDTFVGTYNYMSPERISGSTYDYSSDIWSLGMSVLECAIGRFPYLESEDQQNPPSFYELLAAIVENPPPTAPSDQFSPEFCSFVSACIQKDPPARASSLDLLSHPFIKKFE-DKDIDLGI 343
GhMKK6       RDTFVGTYNYMSPERISGSTYDYSSDIWSLGMVVLECAIGRFPYMQSEDQQSWPSFYELLEAIVEKPPPTAPSDQFSPEFCSFVSACIKKNPKERASSLDLLSHPFIRKFE-DKDIDLGI 341
StMKK6       RDTFVGTYNYMAPERISGSTYDYKSDIWSLGMVILECAIGRFPYIQSEDQQARPSFYELLEAIVSSPPPSAPADQFSPEFCSFVSACIQKDPRDRSSALDLLSHPFIKKFE-DKDIDLSI 341
OsMKK1       RDTFTGTYNYMAPERISGQKHGYMSDIWSLGLVMLELATGEFPYPPRE------SFYELLEAVVDHPPPSAPSDQFSEEFCSFVSACIQKNASDRSSAQILLNHPFLSMYD-DLNIDLAS 331
ZmMKK1       RDTFIGTRNYMAPERIDGKKHGSMSDIWSLGLVILECATGIFPFPPCE------SFYELLVAVVDQPPPSAPPDQFSPEFCGFISACLQKDANDRSSAQALLDHPFLSMYD-DLHVDLAS 333
AtMKK1       ANSFVGTYPYMSPERISGSLYSNKSDIWSLGLVLLECATGKFPYTPPEHKKGWSSVYELVDAIVENPPPCAPSNLFSPEFCSFISQCVQKDPRDRKSAKELLEHKFVKMFE-DSDTNLSA 340
AtMKK2       ANTFVGTYNYMSPERIVGNKYGNKSDIWSLGLVVLECATGKFPYAPPNQEETWTSVFELMEAIVDQPPPALPSGNFSPELSSFISTCLQKDPNSRSSAKELMEHPFLNKYD-YSGINLAS 351
BnMKK1       ANTFVGTYNYMSPERIVGNKYGNKSDIWSLGLVVLECATGKFPYLPPDEEETWSSVLELMEAIVDQPPPTLPSESFSPELSSFISTCLQKDPDSRSSARELMEHPFVKKYDNNSEINLAS 343
PeMKK2a      ANTFVGTYNYMSPERISGGRYDYKSDIWSLGLVLLECATGEFSITPPVPNEGWTNVYELMVAIVDQPPPSAPPDQFSPEFCSFISACVQKDPKDRQSAHELMEHPFMNMYE-DQHVDLSS 344
TEA024893.1  ANTFVGTYNYMSPERICGSKYGSRSDIWSLGLVLLECATGHFPYSPPQQGEGWTNVYELMETIVDQPPPCAPNNQFSPEFCSFISACLKKEPKDRQTANDLMMHPFIKMFK-GVDIDLAS 342
StMKK1       ANTFVGTYNYMSPERISGGAYDYKSDIWSLGLVLLECATGHFPYTPPEGDEGWVNVYELMETIVDQPEPCAPPDQFSPQFCSFISACVQKHQKDRLSANELMSHPFITMYD-DQDIDLGF 342
NbSIPKK      ANTFVGTYNYMSPERILGGAYGYRSDIWSLGLVLLECATGVFPYSPPQADEGWVNVYELMETIVDQPAPSAPPDQFSPQFCSFISACVQKDQKDRLSANELMRHPFITMYD-DLDIDLGS 339
GhMKK1       ANTFVGTYNYMSPERIIGDKYGSKSDIWSLGLVLLECATGQFPYSPPDQAEGWTNFYELMEQIVEQPPPSAPSDRFSSEFCSFISACVKKDPNERKSAHELLGLPFLNMYC-D--VDLES 346

OsMKK6       LVESLEPPMNISE-------- 355
AtMKK6       LVGTLEPPVNYLR-------- 356
GhMKK6       LVGSLEPPVNYPR-------- 354
StMKK6       LVSSLEPPVNFPR-------- 354
OsMKK1       YFTTDGSPLATFNTSNRYDDR 352
ZmMKK1       YFTTAGSPLATFNSRQL---- 350
AtMKK1       YFTDAGSLIPPLAN------- 354
AtMKK2       YFTDAGSPLATLGNLSGTFSV 372
BnMKK1       YFTNAGSPLATLKNLSGTFSV 364
PeMKK2a      YFTNAGS-LATL--------- 355
TEA024893.1  YFTNAGSPLATF--------- 354
StMKK1       YFTSAGPPLATLTEL------ 357
NbSIPKK      YFTSAGPPLATLTEL------ 354
GhMKK1       YFKSAGSPLATLELV------ 361
```

Group B

```
                                                                                                                          GxxGxG
OsMKK3    MAGLEELKKKLQPLLFDDPDKGGVSSRVPLPEDTCDSYVVSDGGTVNLLSRSLGEYNINEHGFHKRSTGP-EESDSGEKAYRCASHDMHIFGPIGNGASSVVQRAVFIPVHRILALKKIN 119
TaMKK3-a  MSGLEELKKKLQPLLFDDTDKGGVSTRVPFPEDTCDSYVVSDGGTINLLSRSFGEYNINEHGFHKRSTGP-EEPDSGEKAYRCASEDMHIFGPIGNGASSVVQRAIFIPVHRILALKKIN 119
ZmMKK3    MAGLEELKKKLQPLLFDDLDRVGASTRVPLPEDTCDSYAVSDGGTVNLLSRSLGEYKINEHGFHKRSAGP-DELDSDEKAYRCASHEMHIFGPIGNGASSVVERAIFIPVHRILALKKIN 119
GhMKK16   MSGLEEFRKKLAPLF--DAEKGFSAGSTLDPYDS---YMLSDSGTVNLLSRSYGVYNINELGLQKCTSSPVDETDQGEKTYRCASHEMRIFGAIGSGASSVVQRAMHIPSHRILALKKIN 115
AtMKK3    MAALEELKKKLSPLF--DAEKGFSSSSSLDPNDS---YLLSDGGTVNLLSRSYGVYNFNELGLQKCTSSHVDESESSETTYQCASHEMRVFGAIGSGASSVVQRAIHIPNHRILALKKIN 115

                                                                                                                DΨK
OsMKK3    IFEKEKRQQILNEMRTLCEACCYIGLVEFQGAFYMPDSGQISIALEYMDGGSLANVIKIKKSIPEPVLAHMLQKVLLGLRYLHEVRHLVHRDIKPANLLVNLKGEAKITDFGVSAGLDNT 239
TaMKK3-a  IFEKEKRQQILNEMRTLCEASCYPGLVEFQGAFYMPDSGQISIALEYMDGGSLADVIKVKKSIPEPVLAHMLQKVLLGLKYLHEVRHLVHRDLKPANLLVKLKGEAKITDFGVSAGLDNT 239
ZmMKK3    IFEKEKRQQILNEMRTLCEACCYPGLVEFQGAFYMPDSGQISIALEYMDGGSLADVIRVKKSIPEPVLSHMLQKVLLGLRYLHEVRHLVHRDIKPANLLVNLKGEAKITDFGVSAGLDNT 239
GhMKK16   IFEKEKRQQLLTEIRTLCEAPCSEGLVEFHGAFYSPDSGQISIALEYMDGGSLADILRLKKSIPEPVLSSMFQKLLQGLSYLHGVRHLVHRDIKPANLLVNLKGEPKITDFGISAGLDNS 235
AtMKK3    IFEREKRQQLLTEIRTLCEAPCHEGLVDFHGAFYSPDSGQISIALEYMNGGSLADILKVTKKIPEPVLSSLFHKLLQGLSYLHGVRHLVHRDIKPANLLINLKGEPKITDFGISAGLENS 235

OsMKK3    MAMCATFVGTVTYMSPERIRNENYSYAADIWSLGLAILECATGKFPYNVNEGPANLMLQILDDPSPTPPKDSYSSEFCSFINDCLQKDADARPSCEQLLSHPFIKRYENTTVDLVAYVKS 359
TaMKK3-a  MAMCATFVGTVTYMSPERIRNENYSYAADIWSLGLTILECATGKFPYNVNEGPANLMLQILDDPSPTPPEDAYTPEFCSFINDCLRKDADARPTCEQLLSHAFIKRYEQTGVDLAAYVRG 359
ZmMKK3    MAMCATFVGTVTYMSPERIRNENYSYAADIWSLGLTILECATGKFPYDVNEGPANLMLQILDDPSPTPPVDTCSLEFCSFINDCLQKDADARPTCEQLLSHPFIKRYAGTEVPLAAYVKS 359
GhMKK16   MAMCATFVGTVTYMSPERIRNESYSYPADIWSLGLALFECGTGEFPYMANEGPVNLMLQILDDPSPSPPKHKFSPEFCSFIDACLQKDADARPTAEQLLSHPFIKKHEHDTVDLAAFVRS 355
AtMKK3    MAMCATFVGTVTYMSPERIRNDSYSYPADIWSLGLALFECGTGEFPYIANEGPVNLMLQILDDPSPTPPKQEFSPEFCSFIDACLQKDPDARPTADQLLSHPFITKHEKERVDLATFVQS 355

OsMKK3    IVDPTERLKQIAEMLAVHYYLLFNGTDGIWHYMKTFYMEESTFSFSGNVYVGQSDIFDTLSNIRKKLKGDCPREKIVHVVEKLHCRAHGETGIAIRVSGSFIVGNQFLICGEGLQAEGMP 479
TaMKK3-a  VVNPT-RLKQIAEMLAVHYYLLFNGSEGPWNYMKTFYKEESSFSFSGNVYVGQSAIFDTLSNIRKKLKGDRPREKIVHVVEKLHCRANGETEIAIRVSGSFITGNQFLIFGEGLQAEGMP 478
ZmMKK3    VVDPTDMLKQIAEMLAIHYYLLFNGPDGIWHHMKTFYMEQSTFSFSENVYVGQNEIFDILSNIRKKLKGDRPREKIVHVVEKLHCRANGETGVAIRVSGSFIVGNQFLVCGEGIKAEGMP 479
GhMKK16   VFDPTQRLKDLADMLTIHYYLLFDGPDENWQHARTLYNEDSTFSFSGKQFVGPNDIHAKLSSIRGTLAGDWPPEKLVHVVEKLQCRAHGQDGVAIRVSGSLIIGNQFLICGDGLQVEGMA 475
AtMKK3    IFDPTQRLKDLADMLTIHYYSLFDGFDDLWHHAKSLYTETSVFSFSGKHNTGSTEIFSALSDIRNTLTGDLPSEKLVHVVEKLHCKPCGSGGVIIRAVGSFIVGNQFLICGDGVQAEGLP 475

OsMKK3    SLEELSIDIPSKRVGQFREQFIMEPGSSMGCYYILRQDLYIIQA- 523
TaMKK3-a  SLEEIDIDIPSKRVGQFREQFTVHPGTSMGCYYIAKQDLYIVQS- 522
ZmMKK3    SLDELSIDIPSKRVGQFREQFIMQPGNLMSCYYISKQDLYIIQS- 523
GhMKK16   NFKDLSIDIPSKRMGTFQEQFIMEPGDLIGRYFIAKQELYIIQ-- 518
AtMKK3    SFKDLGFDVASRRVGRFQEQFVVESGDLIGKYFLAKQELYITNLD 520
```

Group C

```
                                                                                                                      Gx
OsMKK4     -------MRPG---GPPSLRAGLQQQQQQQPGTPGRSRRRPDLTLPLPQRDLTSLAVPLPLPLPPSSAPSSTSSSGSSSLGGVP---------TPPNSVGSAPPAPPPLSELERVRRIGS  101
TaMKK5     -------MRPG---GPPNARPGL---QQQQPGTPGRARRRPDLTLPLPQRDLTSLAVPLPLPPPPSSAPSSTSSSGSGSGSGGASSMPMPMSMTPPNSAGSAPPAPPPLGELERVRRVGS  107
AtMKK4     -------MRPI--------QSPP----GVSVPVKSRPRRRPDLTLPLPQRDVS-LAVPLPLPPTSGGSGGSSGSAPSSGG-------------SASSTNTNSSIEAKNYSDLVRGNRIGS   87
AtMKK5     -------MKPI---------QSP---SGVASPMKNRLRKRPDLSLPLPHRDVA-LAVPLPLPPPSSSSSAPASSSA----------------------ISTNISAAKSLSELERVNRIGS   78
BnaA03.MKK ----------------------------------MRRNKRPDLSLPLPNRNVA-LAVPLPLPPPPSSSSALASSSA----------------------ISTNISAAKRLSELERVNRIGS   63
GhMKK4     MLFLNSVNSPSSGEVRPNQHSQPPAVGGSSSANRNRPRRLAELTVPLPQRTVSLLAVPLPLPPASNAARSTCS-------------------------NGNANSKQVNLSEIDRVNRIGS   95
SlMKK2     -------MRPAANSTNAASSMPP----PSSAGQRSRPRRRTDLTLPLPQRDVA-LAVPLPLPPTSSSSSS------------------------------SPLPTPLHFSELERVNRIGS   78
PtMKK4     -------MKPV---QPPPPQPLP---STNRHTNNSRPRRRPDLKLPLPQRDPQ-LAVPLPLPPNSGGSNSSSS---------------------------SSARAQLSFSELDRINRIGS   79
GhMKK5     -------MRPN--------HQPPPSAGGSSSSNKNRPRRRADLTLPLPQRDPS-LAVPLPLPPSSNSAPPASS-------------------------NSNALPQQVNFSELDRVNRIGS   79

           GxxG                                                                                                 DΨK
OsMKK4     GAGGTVWMVRHRPTGRPYALKVLYGNHDDAVRRQITREIAILRTAEHPAVVRCHGMYEQAGELQILLEYMDGGSLEGRRIASEAFLADVARQVLSGIAYLHRRHIVHRDIKPSNLLIDSG  221
TaMKK5     GAGGTVWLVRHAPTGRAYALKVLYGHHDEAVRRQITREIAILRTAEHPSIVRCHGMYEQAGELQILLEYMDGGSLDGRRIASEAFLADVARQVLSGIAYLHRRHIVHRDIKPSNLLIDSG  227
AtMKK4     GAGGTVYKVIHRPSSRLYALKVIYGNHEETVRRQICREIEILRDVNHPNVVKCHEMFDQNGEIQVLLEFMDKGSLEGAHVWKEQQLADLSRQILSGLAYLHSRHIVHRDIKPSNLLINSA  207
AtMKK5     GAGGTVYKVIHTPTSRPFALKVIYGNHEDTVRRQICREIEILRSVDHPNVVKCHDMFDHNGEIQVLLEFMDQGSLEGAHIWQEQELADLSRQILSGLAYLHRRHIVHRDIKPSNLLINSA  198
BnaA03.MKK GAGGTVYKVIHLPTSRPFALKVIYGNHDDNVRRQICREIEILRSVDHPNVVKCHDMFDHNGEVQVLLEFMDKGSLEGRHVSRENELAGLTRQILSGLAYIHRRHIVHRDIKPSNLLINSA  183
GhMKK4     GAGGTVYKVIHRPSSRLYALKVIYGNHEETVRRQIRREIEILRDVNHPNVVKCHEMYDRNGEIQVLLEFMDVGSLEGTHISHESNLSDLARQILNGLNYLHRRHIVHRDIKPSNLLINSK  215
SlMKK2     GTGGTVYKVLHRPTGRLYALKVIYGNHEDSVRLQMCREIEILRDVDNPNVVRCHDMFDHNGEIQVLLEFMDKGSLEGIHIPLEQPLSDLTRQVLSGLYYLHRRKIVHRDIKPSNLLINSR  198
PtMKK4     GSGGTVYKVVHRPTSRLYALKVIYGNHEDSVRNSICREIEILRDVNHPNVVKCHDMFDHNGEIQVLLEFMDGGSLEGTHINHEGYLSDVARQILNGIAYLHKRKIVHRDIKPSNLLINSK  199
GhMKK5     GTGGTVYKVVHRPSSRPYALKVIYGNHEESVRRQIRREIEILRDVDHPNVVKCHEMYDHNGEIQVLLEFMDGGSLEGILISREANLSDLARQVLSGLNYLHRRHIVHRDIKPSNLLTNSK  199

OsMKK4     RRVKIADFGVGRILNQTMDPCNSSVGTIAYMSPERINTDLNDGAYDGYAGDIWSFGLSILEFYMGRFPLGENLGKQGDWAALMCAICY-SDSPAPPNASPEFKSFISCCLQKNPARRPS  340
TaMKK5     RRVKIADFGVGRILNQTMDPCNSSVGTIAYMSPERINTDLNDGNYNGYAGDIWSFGLSILEFYLGRFPLGENLGKQGDWAALMCAICY-SESPAAPPTASPELRSFISCCLQKNPAKRPS  346
AtMKK4     KNVKIADFGVSRILAQTMDPCNSSVGTIAYMSPERINTDLNQGKYDGYAGDIWSLGVSILEFYLGRFPFP--VSRQGDWASLMCAICM-SQPPEAPATASPEFRHFISCCLQREPGKRRS  324
AtMKK5     KNVKIADFGVSRILAQTMDPCNSSVGTIAYMSPERINTDLNHGRYDGYAGDVWSLGVSILEFYLGRFPFA--VSRQGDWASLMCAICM-SQPPEAPATASQEFRHFVSCCLQSDPPKRWS  315
BnaA03.MKK NNVKIADFGVSRILAQTMDPCNSSVGTIAYMSPERINTDLNHGRYDGYAGDIWSLGVSVLEFYLGRFPFA--VSRQGDWASLMCAICM-TQPPEAPATASEDFRHFISCCLQSDPPKRWS  300
GhMKK4     RKVKIADFGVSRILDQTMHPCNSSVGTVAYMSPERINTDLNQGQYDGYAGDIWSLGVSILEFYLGRFPFA--VGRQGDWASLMCAICMSSQPPEAPPTASYEFRHFISCCLQKDPTRRLT  333
SlMKK2     REVKIADFGVSRVLAQTMDPCNSSVGTIAYMSPERINTDLNHGQYDGYAGDIWSLGVSILEFYLGRFPFS--VGRQGDWASLMCAICM-SQPPEAPPSASREFREFIACCLQRDPARRWT  315
PtMKK4     NNVKIADFGVSRILAQTMDPCNSSVGTIAYMSPERINTDLNKGMYDGYAGDIWSLGVSILEFYLGRFPFG--VGRQGDWASLMCAICM-SQPPEAPATASREFRDFIACCLQREPARRFT  316
GhMKK5     KVVKIADFGVSRILDQTMDPCNSSVGTIAYMSPERINTDLNHGLYDGYAGDIWSLGVSILEFYLGRFPFA--VGRQGDWASLMCAICM-SQPPEAPPTASNEFRHFISCCLQRDPARRWS  316

OsMKK4     AAQLLQHRFVA---------GPQQQQQPQPQPLAPPPS----------  369
TaMKK5     AAQLLQHRFIA------------SPPQQQPQVLAAPPC----------  372
AtMKK4     AMQLLQHPFILRAS------PSQNRSPQNLHQLLPPPRPLSSSSSPTT  366
AtMKK5     AQQLLQHPFILK-----------ATGGPNLRQMLPPPRPLPSAS----  348
BnaA03.MKK AQQLLQHPFIVK-----------SSGGPNLRQMLPPPPPAS-------  330
GhMKK4     AAQLLQHPFIIR-------------GQNQVA-----------------  351
SlMKK2     AGQLLRHPFITQNSTGTTHTGPATTTTSLSHPLLPPPPHFSSSS----  359
PtMKK4     ANQLLQHPFIVRRA------GEGSGGNGTHSLMLPPPPRPIP------  352
GhMKK5     AAQLLQHPFILR--------GQPHQVAQNLHQLLPPPPPLSS------  350
```

Group D

```
OsMKK10-2 MALVRQRRHLP--------------------------------HLTLPLDHFALRPPPAPQQQQ-----------------QPAVAPS---------TSSDVRLSDFERISVLGHGNGG  61
AtMKK10   MTLVRERRHQE--------------------------------PLTL--------SIPPLIYHGT-AFSVASSSSS------SPETSPI-------------QTLNDLEKLSVLGQGSGG  60
OsMKK10-1 MAKLRERRQL---------------------------------RLSVP-------ASPPPFPHLDHPFAA-LPS--------TPPGSPV---------------LAELEMLSVVGRGAGG  56
ZmMKK10   MALAGDERLPPFHLSLNVPSRPAVQESSFRHANPPSSTKAIARNKRLPPFHLSLNVPSRPAAQEP-SSRHANPPVAAP----EPASTPL--------ARSTQFRLADFDRLAVLGRGNGG 107
AtMKK8    MVMVRDNQFL---------------------------------NLKLS-------PIQAPTTIPPCRFPI-IPA--------TKVSATV------SSCASNTFSVANLDRISVLGSGNGG  65
AtMKK7    MALVRKRRQI---------------------------------NLRL--------PVPPLSVHLP-WFSF------------ASSTAPV---------INNGISASDVEKLHVLGRGSSG  57
AtMKK9    MALVRERRQL---------------------------------NLRL--------PLPPISDR---RFSTSSS---------SATTTTV--------AGCNGISACDLEKLNVLGCGNGG  59
RhMKK9    MALIRERRQL---------------------------------NLSL--------PLPEPSERRP-RFTLPLPP--------TAAVATT-------TNCSSAFSASDLERLEVLGHGNGG  63
NbMKK5    MALVRERRQL---------------------------------NLRL--------PLPEPSERRP-RFPLPIPPSSICTTNSTANTATT-------TASTTTISISELEKLKVLGHGNGG  71
SlMKK4    MALVRDRRHL---------------------------------NLRL--------PLPEPSERRP-RFPLPLPPSSVPTVNSTASTTTATNTTTTTTASTTTISISELEKLKVLGHGNGG  78
StMKK5    MALVRDRRHL---------------------------------NLRL--------PLPEPSERRP-RFPLPLPPSSVSTVNSTANTTTT--TTTTTTASTTTISISELEKLKVLGHGNGG  76

OsMKK10-2 TVYKARHRRGCPAQQPLALKLFAAGDLSA------AREAEILRLAADA----PHVVRLHAV-VPSAAGGVEEPAALALELMPGGSLAGLLRRLGRPMGERPIAAVARQALLGLEALHALR 170
AtMKK10   TVYKTRHRRTKTL---YALKVL-RPNLNTTV----TVEADILKRIE-S----SFIIKCYAV-FVSLYD-----LCFVMELMEKGSLHDALLAQQ-VFSEPMVSSLANRILQGLRYLQKMG 160
OsMKK10-1 TVYRARHRRTGAA---LAVKEM-RDDGAA------LREAGAHLRVAAAAPDHPSVVRLHGVCVGHPVAGN-RFVYLVLEYLPEGSLSDVLV-RG-ALPEPAIAGVTRCVLRGLSHLHRLG 163
ZmMKK10   TVYKVRHRETCAL---YALKVL-HEDAGA--------EADILGRLA-S----PFVVRCHAVLPASCSAGD---VALLLELVDGGSLDAVSRRRG-AFAEAALAEVAAQALSGLAYLHARR 206
AtMKK8    TVFKVKDKTTSEI---YALKKV-KENWDSTS----LREIEILRMVN-S----PYVAKCHDI-FQNP-SGE---VSILMDYMDLGSLESL----R-GVTEKQLALMSRQVLEGKNYLHEHK 162
AtMKK7    IVYKVHHKTTGEI---YALKSV-NGDMSPAFTRQLAREMEILRRTD-S----PYVVRCQGI-FEKPIVGE---VSILMEYMDGGNLESL---RG-AVTEKQLAGFSRQILKGLSYLHSLK 160
AtMKK9    IVYKVRHKTTSEI---YALKTV-NGDMDPIFTRQLMREMEILRRTD-S----PYVVKCHGI-FEKPVVGE---VSILMEYMDGGTLESL---RG-GVTEQKLAGFAKQILKGLSYLHALK 162
RhMKK9    TVYKVRHKRTGAN---YALKIV-KGDSDPTVRRQLYREIEILRRTD-S----PHVVHCNAI-IEKP-TGD---IWILMEYMDSGTLETSLKENG-TFSEAKVARVARQVLNGLNYLHTQK 168
NbMKK5    TVYEVRHKRTSAI---YALKVV-HGDSDPEIRRQILREISILRRTD-S----PYVIKCHGV-IDMP-GGD---IGILMEYMNVGTLESLLKSQA-TFSELSLAKIAKQVLSGLDYLHNHK 176
SlMKK4    TVYKVRHKRTSAI---YALKVV-HGDSDPEIRRQILREISILRRTE-S----PYVIKCHGV-IDMP-GGD---IGILMEYMNAGTLENLLKSQL-TFSELCLARIAKQVLGGLDYLHSHK 183
StMKK5    TVYEVRHKRTSAI---YALKVV-HGDSDPEIRRQILREISILRRTD-S----PYVIKCHGV-VDMP-GGD---IGILMEYMNAGTLENLLKSQS-TFSELCLAKIAKQVLGGLDYLHSHK 181

             DΨK
OsMKK10-2 IVHRDLKPSNLLLGADGEVKIADFGAGKVLRRRLDPCASYVGTAAYMSPERFDPEAYS----GDYDPYAADVWSLGVAILELYLGHFPLLPVGQRPDWAALMCAICFGEAPEMPAA-ASE 285
AtMKK10   IVHGDIKPSNLLINKKGEVKIADFGASRIVAGGDYGSN---GTCAYMSPERVDLEKWGF---GGEVGFAGDVWSLGVVVLECYIGRYPLTKVGDKPDWATLFCAICCNEKVDIPVS-CSL 273
OsMKK10-1 VAHGDVKPSNLLVGHRGEIKIADFGASRVVTGRDEAHHQSPGTWAYMSPEKLHPEGFGG---GGGADFSGDVWSLGVVLLECHAGRFPLVAAGERPDWPALVLAVCFAAAPEVPVA-ASP 279
ZmMKK10   VVHLDVKPSNLLATAAGEIKVADFGIARVLSRSGDHCTSYVGTAAYMSPERFDPEAHG----GHYDPCAADVWSLGVTVLELLMGRYPLLPAGQQPNWAALMCAICFGEPPALPDGAASP 322
AtMKK8    IVHRDIKPANLLRSSKEEVKIADFGVSKIVVRSLNKCNSFVGTFAYMSPERLDSEADGVTEEDKSNVYAGDIWSFGLTMLEILVGYYPML-----PDQAAIVCAVCFGEPPKAPEE-CSD 276
AtMKK7    IVHRDIKPANLLLNSRNEVKIADFGVSKIITRSLDYCNSYVGTCAYMSPERFDSAA-G----ENSDVYAGDIWSFGVMILELFVGHFPLLPQGQRPDWATLMCVVCFGEPPRAPEG-CSD 274
AtMKK9    IVHRDIKPANLLLNSKNEVKIADFGVSKILVRSLDSCNSYVGTCAYMSPERFDSESSG----GSSDIYAGDIWSFGLMMLELLVGHFPLLPPGQRPDWATLMCAVCFGEPPRAPEG-CSE 277
RhMKK9    IIHRDIKPANLLVNSNMEVKIADFGVSKMMCRTLDNCNSYVGTCAYMSPERFDPDTYG----GNYNGYASDIWSLGLTLMELYMGHFPFLPPGQRPDWATLMCAICFGEPPALPEG-VSE 283
NbMKK5    IIHRDLKPSNLLVNREMEVKIADFGVSKIMCRTLDPCNSYVGTCAYMSPERFDPGTYG----VNYNGYAADIWSLGLTLMELYIGHFPFLPPGQRPDWATLMCAICFGEPPSLPEG-TSV 291
SlMKK4    IIHRDLKPSNLLVNREMEVKIADFGVSKIMGRTLDPCNSYVGTCAYMSPERFDPDTYG----GNYNGYAADIWSLGLTLMELYMGHFPFLPPGQRPDWATLMCAICFGEPPSLPEN-TSE 298
StMKK5    IIHRDLKPSNLLVNREMEVKIADFGVSKIMGRTLDPCNSYVGTCAYMSPERFDPDTYG----ANYNGYAADIWSLGLTLMELYMGHFPFLPPGQRPDWATLMCAICFGEPPSLPEN-TSE 296

OsMKK10-2 EFRDFVSRCLEKKAGRRASVGELLEHPFIAERDAADAQRSLAALVAEAEQSGDL------- 339
AtMKK10   EFRDFVGRCLEKDWRKRDTVEELLRHSFVKNR----------------------------- 305
OsMKK10-1 EFGGFVRRCLEKDWRRRATVEELLGHPFVAGKPSRCERQNEWRTTFQDKTGQVNTSIYGEE 340
ZmMKK10   ELRSFISACLHKDYCRRASVAELLAHPFIVGRDVLASRDALQQLVAEA------------- 370
AtMKK8    DLKSFMDCCLRKKASER-------------------------------------------- 293
AtMKK7    EFRSFVDCCLRKESSERWTASQLLGHPFLRESL---------------------------- 307
AtMKK9    EFRSFVECCLRKDSSKRWTAPQLLAHPFLRE-DL--------------------------- 310
RhMKK9    EFRSFMECCLQKESGKRWTAAQLLTHPFVCK-DPKAIRYLKD------------------- 324
NbMKK5    NFRDFIECCLQKESSKRWSAQQLLNHPFILSNLK--------------------------- 325
SlMKK4    KFNDFMKCCLQKESSKRWSAHQLLQHPFIQSIDLKST------------------------ 335
StMKK5    KFNDFMKCCLQKESSKRWSAHQLLQHPFIQSIDLKST------------------------ 333
```

Figure S1 Alignment of domains in MKKs of plant. All the MKK protein sequences were aligned by MAFFT v7.0. Sequences of additional MKK domains that are highlighted are ATP binding signature, consisting the P loop consensus sequence (GxGxxG), marked in cyan, the catalytic C loop, consisting the DΨK consensus, marked in orange colour, the activation T loop, marked in green colour and NTF2 domain marked in pink colour. The sequence derivations from the S/TxxxxxS/T activation loop of Clade C and D are marked in a lighter shade of green colour.
